# Study on the Material Removal Mechanism of Ultrasonic Elliptical Vibration Cutting of Medical β Titanium Alloy

**DOI:** 10.3390/mi13060819

**Published:** 2022-05-25

**Authors:** Zhenda Wang, Yongzhi Pan, Yijia Zhang, Xiuhua Men, Xiuli Fu, Shengfeng Ren

**Affiliations:** 1 School of Mechanical Engineering, University of Jinan, Jinan 250024, China; 202021100286@stu.ujn.edu.cn (Z.W.); me_panyz@ujn.edu.cn (Y.P.); 202121100307@stu.ujn.edu.cn (Y.Z.); me_menxh@ujn.edu.cn (X.M.); me_rensf@ujn.edu.cn (S.R.); 2 Linqing Institute of industry and Technology of Shandong, Liaocheng 252600, China

**Keywords:** medical β titanium alloy, ultrasonic elliptical vibration cutting, composite cutting process, material removal mechanism

## Abstract

For new medical β titanium implants, the surface micro texture processing technology is a difficult problem. To solve this problem, a new method of ultrasonic elliptical vibration cutting (UEVC) is adopted in this paper. The mechanism of material removal in ultrasonic elliptical vibration cutting is explored for different cutting paths. By means of simulation and experimentation, the material removal mechanism of ultrasonic elliptical vibration cutting medical β titanium alloy is revealed with respect to the aspects of cutting deformation, stress distribution, force and thermal variation, and chip formation mechanism. The results show that: (1) The cutting temperature and cutting force in the UEVC process obey the law of periodic change, and the maximum point of cutting force appears ahead of the maximum point of cutting temperature. (2) The material removal process of UEVC is a “press–shear–pull” composite cutting process. The tool squeezes the material to form the chips. Under the action of high temperature, the material is removed by adiabatic shear. (3) The difference of UEVC paths will affect the removal mode of materials and form different surface morphology. (4) For different cutting paths, compressive stress is distributed at the lowest point of the machining pit, and tensile stress is distributed at the protrusion position.

## 1. Introduction

The demand for medical implants such as bone implantations and bone replacements is increasing due to joint diseases and the aging population. Titanium alloy has the characteristics of being non-magnetic, corrosion resistant, and possessing high strength and high toughness. In particular, metastable β titanium alloy is a new type of medical titanium alloy. Its elastic modulus is similar to that of human bones. It can effectively avoid the problem of stress shielding. In addition, the material does not contain cytotoxic elements. Therefore, it is favored in the medical field [1,2].

To improve the biocompatibility and wear resistance of medical titanium implants, micro texture on the surface is usually processed in order to realize surface modification [3,4]. At present, laser engraving, acid etching, alkali etching, and other methods are used to process the surface micro texture of titanium implants, but it is difficult to accurately control the geometry and surface morphology of the micro texture [5,6,7]. Moreover, micro textured surfaces processed by laser are prone to slag, irregular shape, and complex residual stress [8,9,10]. Therefore, UEVC can be used to solve the problems of difficult machining and unstable surface texture processing quality in titanium implants. It also overcomes the problems of high cutting temperature, heavy tool wear, and poor machining quality in the traditional cutting process [11,12,13,14].

UEVC was first proposed by the Japanese scholars Shamoto Eiji and Moriwaki Toshimichi [15]. By applying ultrasonic excitation in two or more directions of the tool, the tool tip can cut the workpiece along an elliptical path. In this paper, ultrasonic excitation is applied in the cutting direction and cutting depth direction of the tool in order to process the pit texture on the surface of the workpiece. Lotfi et al. [16] used UEVC to texture the surface of titanium alloy. It was found that micro texture was formed on the surface of the material under the impact of certain frequencies. Friction and wear tests were carried out. The results showed that the surface micro texture could effectively improve the friction and wear properties of titanium alloy. Zhang et al. [17] processed sinusoidal, sawtooth, oblique wave and other different groove nanostructures on hardened steel by means of amplitude control in ultrasonic elliptical vibration cutting. The restrictions of vibration conditions and tool geometry on machining shape were studied, and a compensation method for amplitude control command was proposed. Yang et al. [18] prepared ordered micro/nano grating structures on the surface of aluminum alloy using the ultrasonic elliptical vibration cutting process. The influence mechanism of micro texture on surface color and optical reflectivity was studied theoretically and experimentally.

The above research verified the machinability of UEVC in the processing of material surface micro texture. However, the high-quality processing of micro texture still needs to be deeply studied with respect to its mechanism, exploring the stress state, removal mode, chip shape and so on. Ma et al. [19] studied the effect of diamond tools on the critical cutting depth of brittle materials under the condition of ultrasonic vibration by performing groove cutting tests on brittle materials. It was found that under the condition of ultrasonic vibration, diamond tools can increase the critical cutting depth for the plastic cutting of brittle materials. Liu et al. [20] conducted molecular dynamics simulation using the improved model to explore the material removal mechanism of monocrystalline silicon under EVC. The results showed that the main material removal mechanism shifts from extrusion to shear in one vibration cycle. In addition, based on stress analysis, it was found that the formation mechanism of subsurface damage in the extrusion and shear stages is different. Huang et al. [21] developed a ductile zone machining model for UEVC of brittle materials based on the plastic zone machining model with the aim of achieving the maximum cutting depth, so as to maximize the machining efficiency while ensuring the machining surface quality. Liu et al. [22] studied the effect of amplitude on machined surface integrity in high-speed ultrasonic elliptical vibration milling of titanium alloy. It was found that the surface roughness increased with increasing vibration amplitude, and the surface residual compressive stress increased with increasing vibration amplitude. Gao et al. [23] used ultrasonic elliptical vibration milling to effectively improve the quality of the machined surface. The research found that, compared with ordinary milling, high-speed ultrasonic vibration milling demonstrated a stable improvement in the tool yield and surface roughness of the machined surface. The above research on the cutting mechanism of UEVC materials mostly focused on a single material removal method. Moreover, there is still a lack of theoretical research on micro texture processing of β titanium implants, which is a difficult-to-machine material.

This paper focuses on the technical problems of β titanium implant processing. The UEVC processing method is adopted. With the help of finite element simulation, the evolution laws of chip morphology, residual stress, and maximum principal stress in the machining process under different cutting trajectories are explored. The influence mechanism of the material removal process is revealed, and a complete model of the material removal process of UEVC is established. This provides theoretical guidance for the processing of the micro texture on the surface of β titanium implants.

## 2. UEVC Theoretical Model

### 2.1. Kinematic Model

UEVC is realized by applying periodic ultrasonic excitation with the same frequency and different amplitude to the cutting direction and cutting depth direction of the tool. Finally, the machining of micro texture on the workpiece surface is realized.

**Figure 1:**
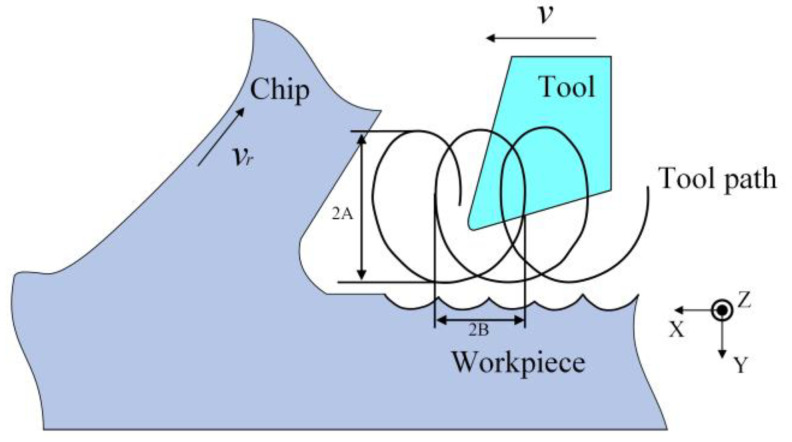
Schematic diagram of UEVC.

As shown in Figure 1, ultrasonic excitation is applied in the X direction (cutting speed direction) and the Y direction (cutting depth direction) to establish a UEVC process under ideal conditions.

The tool tip trajectory equation is as follows:

(1) x(t)=Asin(2πft+φ)

(2) y(t)=Bcos(2πft)

With the cutting speed, the trajectory equation of the tool tip relative to the workpiece is as follows:

(3) x(t)=Asin(2πft+φ)+vt

(4) y(t)=Bcos(2πft)

where *A* and *B* are the amplitudes in X and Y directions, respectively. *f* is the ultrasonic vibration frequency, which is 20 kHz in this paper. *φ* is the phase difference of two-way sinusoidal excitation. *v* is the cutting speed.

The speed of the tool tip relative to the workpiece can be derived from Equations (3) and (4):

(5) $$v_{x}\left(t\right)=2\pi fAcos\left(2\pi ft+\varphi \right)+v$$

(6) $$v_{y}\left(t\right)=-2\pi fBsin\left(2\pi ft\right)$$

Different cutting paths can be obtained by adjusting the parameters (*A*, *B*, *f*, *φ* and *v*) according to the above formula. According to Formulas (5) and (6), if $v_{x}\left(t\right)$ ≥ 0 at any time *t*, the tool and workpiece will not be separated, which is called non-separated ultrasonic vibration cutting. If *t* makes $v_{x}\left(t\right)$ < 0, there will be a separation stage between the tool and the workpiece, which is called separated ultrasonic vibration cutting [24]. Aiming at the high-quality processing of β titanium implant surface micro texture, this paper only explores the process of separated UEVC.

### 2.2. Cutting Path Planning

**Figure 2:**
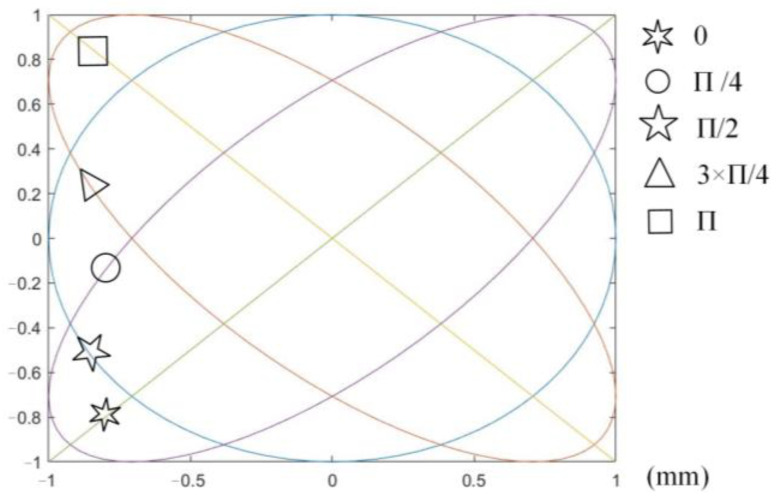
Single-cycle cutting trajectory.

Compared with traditional cutting, UEVC has more flexible trajectory control and more prominent advantages in the processing of material surface micro texture. Different cutting paths can be generated by adjusting the control parameters of cutting path (vibration frequency, phase difference, amplitude and cutting speed). Finally, micro textures with different shapes are processed on the material surface. The single-period trajectory can be obtained by adjusting the phase difference and amplitude using MATLAB software, as shown in Figure 2.

**Figure 3:**
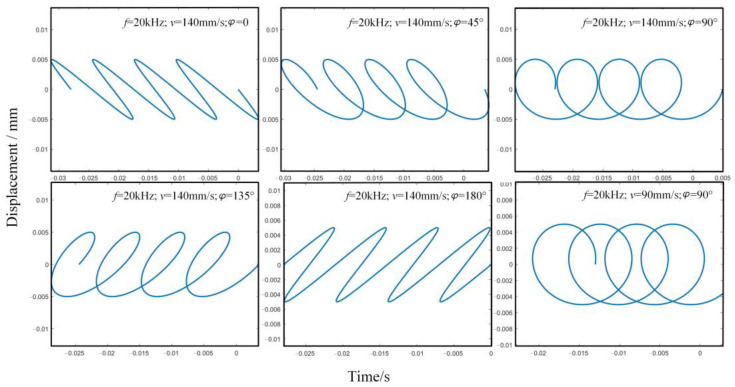
Variation of cutting trajectory under different parameters.

The single-cycle trajectory parameters are adjusted in combination with the cutting speed to obtain the multi-cycle cutting trajectory under different parameters, as shown in Figure 3. In the figure, *A* and *B* in Equations (5) and (6) are taken as 0.005.

### 2.3. Construction of UEVC Simulation Model

Limited by experimental conditions such as ultrasonic equipment and external environment, the actual cutting trajectory obtained by adjusting parameters such as excitation voltage and frequency is often different from the theoretical trajectory. In addition, the experiment is difficult to reflect the laws of material surface stress and strain, chip formation and temperature evolution in the whole process of UEVC, so it is impossible to accurately explore the material removal mechanism in the whole process of UEVC. It is difficult to give full play to the unique advantages of UEVC. Therefore, finite element simulation is used to simulate the planned theoretical trajectory. The cutting force, cutting temperature, stress evolution and surface morphology in the cutting process are explored to reveal the UEVC material removal mechanism of medical β titanium alloy.

**Figure 4:**
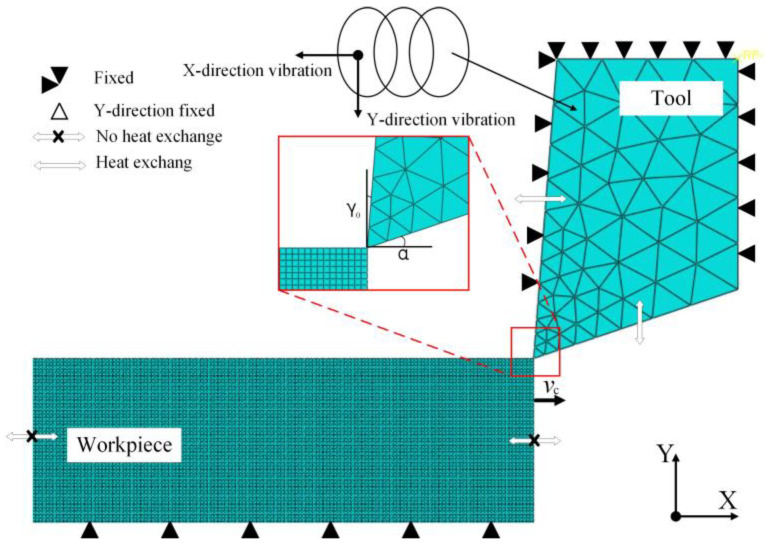
2D-UEVC simulation model.

ABAQUS software is used to establish a 2D UEVC model, as shown in Figure 4. The amplitude and cutting depth of the UEVC process are at the micron level, and the ultrasonic frequency is set to 20 kHz. Therefore, micro-machining is used in the simulation process to more intuitively observe the material removal mode in the cutting process. The workpiece is made of metastable β titanium alloy with a size of 100 × 55 μm. The tool material is a single crystal diamond. In Figure 4, *γ*_0_ is 5° and α is 15°. To obtain better texture morphology, and because the cutting edge of single crystal diamond tool is very sharp, the blunt circle radius of its cutting edge reaches nanometer level. Therefore, the tool model in the simulation ignores the influence of the blunt circle radius of the cutting edge. ALE (Arbitrary Lagrangian Eulerian adaptive meshing) adaptive method is used to divide the mesh to reduce the error caused by excessive distortion of the element mesh in the simulation process. The single precision offset method is used to mesh the rake face and flank of the tool. The workpiece mesh type adopts CPE4RT (four-node plane strain thermally coupled quadrilateral element) and the tool mesh type adopts CPE3T (three-node plane strain thermally coupled triangular element). The mesh size of the upper part of the workpiece is 0.0003 × 0.0003 mm. The mesh size of the lower part is 0.003 × 0.0003 mm. The lower part of the workpiece does not contact with the tool, so the lower part of the mesh adopts a large size to reduce the amount of calculation. The lower left corner of the model is taken as the reference point, and the lower half and bottom of both sides of the model are constrained in a completely fixed way.

β titanium alloy is an elastic–plastic material, so the J-C (Johnson–Cook) constitutive model with simple form and convenient solution is adopted [25]. Because of the introduction of parameters such as the strain strengthening and strain rate strengthening of plastic materials, the dynamic behavior of materials in the cutting process can be accurately described. By analyzing the mesh deformation and stress distribution of materials, the material cutting process is accurately described. The mathematical expression is as follows:

(7) $$\sigma =\left(A+B\varepsilon^{n}\right)\left(1+C\ln\dot{\varepsilon }\right)\left[1-{\left(\frac{T-T_{r}}{T_{m}-T_{r}}\right)}^{m}\right]$$

Of which T, $T_{r}$ and $T_{m}$ are deformation temperature, room temperature and material melting point, respectively. *A* is the initial yield stress. *B* is the strain hardening modulus. *n* is the hardening index. *C* is the strain rate sensitivity coefficient. *m* is the thermal softening index. σ is flow stress. ε is strain. $\dot{\varepsilon }$ is the strain rate. The fracture failure criterion of β titanium alloy materials adopts J-C shear failure cumulative failure criterion.

The definition unit of the J-C fracture failure damage model is as follows:

(8) $$d=\sum \frac{△\varepsilon p}{\varepsilon f}$$

where *d* is the failure parameter, *d* = 0–1, initially *d* = 0, when *d* = 1, the material fails. △εp is the plastic strain increment of one time step. εf is the failure strain of the current time step. Failure strain εf is as follows:

(9) $$\varepsilon f=\left(d_{1}+d_{2}exp(d_{3}\sigma^{*}\right))\left(1+d_{4}\ln\varepsilon^{*}\right)\left(1+d_{5}T^{*}\right)$$

where *d*_1_, *d*_2_, *d*_3_, *d*_4_ and *d*_5_ are material parameters. $\sigma^{*}$ is stress triaxiality. $\varepsilon^{*}=\varepsilon /\varepsilon_{0}$ is the dimensionless plastic strain rate and $\varepsilon_{0}$ is the reference plastic strain rate. $T^{*}$ is dimensionless temperature.

In addition, in order to get better simulation results of chip and cutting force, the friction model in the model is set reasonably. To conform to the sliding friction form between chip and tool surface in the actual cutting process, the friction form in the simulation is defined by Coulomb model. The formula is as follows:

(10) $$\tau_{f}=\mu \sigma_{n}$$

where $\tau_{f}$ is the friction stress. μ is Coulomb friction coefficient. $\sigma_{n}$ is the normal compressive stress in the contact area.

Based on the established simulation model, the workpiece parameters are set as shown in Table 1. In the table, K represents the coefficient of thermal conductivity of the workpiece and *C* represents the specific heat of the workpiece.

**Figure 5:**
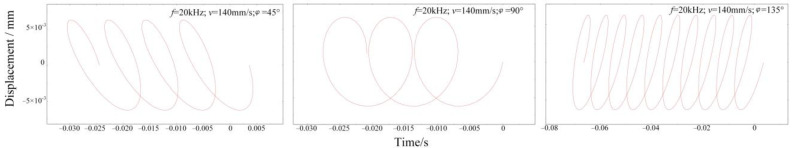
Simulation of cutting path.

The trajectory parameters are extracted in MATLAB. The extracted parameters are set as the eigenvalues of the periodic function in X and Y directions in ABAQUS, and the velocity periodic function in X and Y directions is established. The cutting path is generated in ABAQUS, as shown in Figure 5. To explore the removal mode of UEVC material under different cutting trajectories, this paper simulates and analyzes the three greatly different cutting trajectories shown in Figure 5. In the figure, *A* and *B* in Equations (5) and (6) are taken as 0.005.

## 3. UEVC Experimental Verification and Analysis

### 3.1. UEVC Experiment

**Figure 6:**
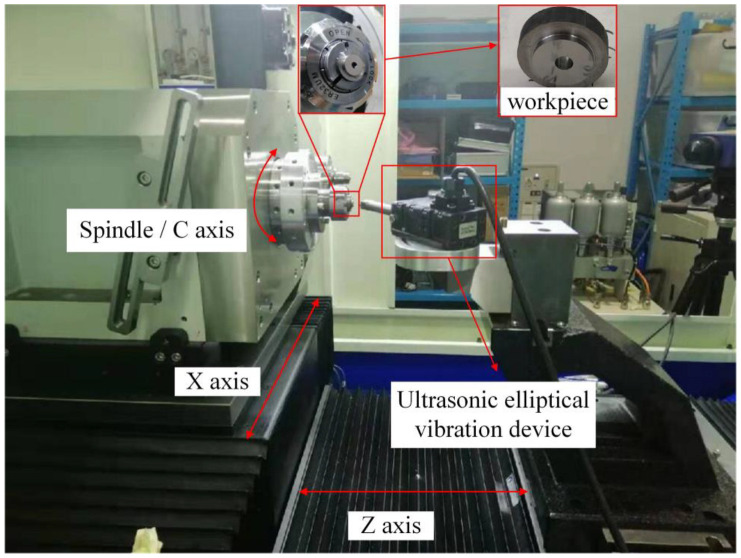
Schematic diagram of cutting device and workpiece.

The experimental material is a new medical titanium alloy represented by metastable β titanium alloy (Ti-25Nb-10Ta-1Zr-0.2Fe). It has the characteristics of high plasticity, high elasticity, and high strength, and has good biomedical properties [2,26]. The cutting device adopted is the Taga ultrasonic elliptical vibration cutting equipment in Japan. It was installed on the MQ-350 two-axis precision lathe for UEVC experiment. Oil cooling is used to cool and lubricate the cutting area. The workpiece is a ring sample, one end of which is fixed to the three-jaw chuck of the lathe, and its end face is processed by UEVC. The schematic diagram of cutting device and workpiece is shown in Figure 6. A single crystal diamond turning tool is selected as the UEVC tool. The rake angle of the tool is 5°, the back angle is 15°, the arc radius of the tool tip is 1 mm, and the blunt radius of the cutting edge is 0.04 μm. The experimental parameters are shown in Table 2.

Machining surface measurement: The surface morphology and roughness of the machined workpiece are measured by WLI-NV5000 5022S. Five areas are taken from each processed sample for inspection. The average value of five areas is taken as the surface roughness value under this machining parameter.

### 3.2. Analysis of UEVC Surface Topography

**Figure 7:**
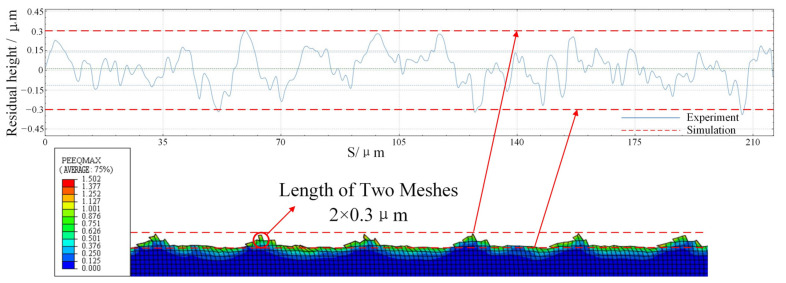
Comparison of machining residual height between experiment and simulation.

**Figure 8:**
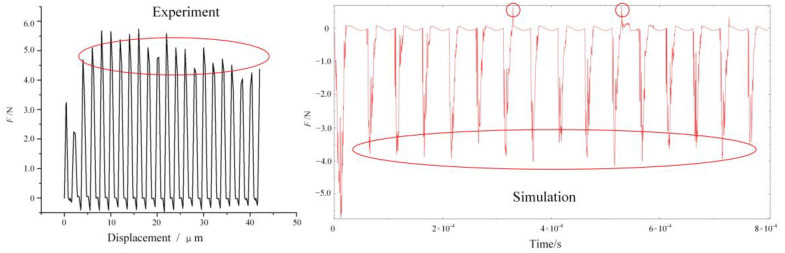
Comparison of cutting force between experiment and simulation.

Figure 7 shows a comparison of the residual height of the machined surface between UEVC experiment and simulation under the same parameters. The dotted line in the figure is the upper and lower limits of the residual height obtained by simulation, and the solid line is the surface residual height obtained by experiment. The residual height obtained by simulation can be extracted by the query function in the software, and can also be obtained by calculating the number of meshes in the figure. Because the tool model in the simulation does not consider the blunt circle radius of the cutting edge, the residual height obtained by the experiment is generally within the upper and lower limits of the residual height obtained by the simulation. Due to the errors in the experiment and the influence of machine tool vibration, the residual height obtained in the experiment fluctuates up and down in a certain range. However, the overall fluctuation trend is within the limits of the simulation results. Combined with the cutting force comparison between the experiment and simulation process under the same parameters as shown in Figure 8, it is considered that the established UEVC simulation model has high accuracy. It can be seen from Figure 8 that the error of cutting force extracted by experiment and simulation is within 20%. The cutting force obtained from simulation and experiment has periodic characteristics. In the stage of tool–workpiece separation, the cutting force is 0.

**Figure 9:**
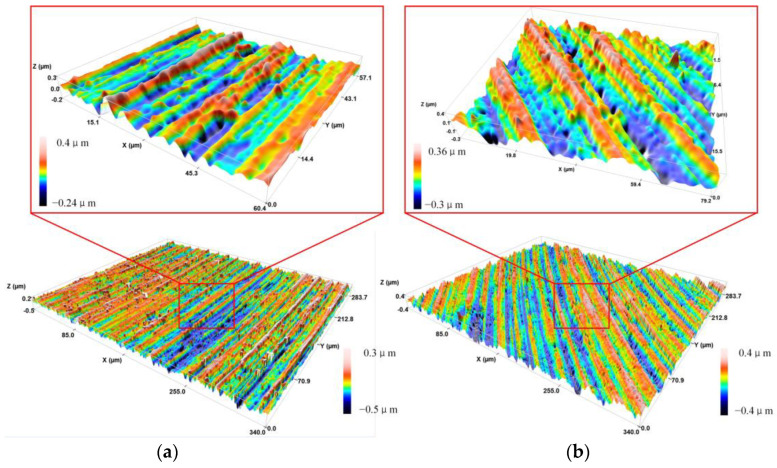
Three-dimensional surface morphology of UEVC workpiece. (**a**) The feed rate is 20 μm/r; (**b**) the feed rate is 25 μm/r.

Figure 9 shows the three-dimensional surface morphology of an area of UEVC workpiece, and the feed rate of Figure 9a is 20 μm/r, the feed rate of Figure 9b is 25 μm/r, the other parameters are consistent. It can be seen from the figure that there are evenly arranged pits on the workpiece surface processed by UEVC along the cutting direction. According to the analysis of the UEVC cutting characteristics, the pit is the result of the material removal by the periodic vibration of the tool along the cutting depth direction. By measuring the surface roughness of the workpiece, it is found that when the feed rate is 20 μm/r, the surface roughness of the material is much lower than that of the workpiece with the feed rate of 25 μm/r. The main reason for this is that when the feed rate is too large, the plowing effect of the tool on the material is more obvious. Due to the large amount of material removal and high temperature in the cutting area, the effect of UEVC on inhibiting furrow and scale thorn is gradually weakened. At the same time, with increasing cutting depth, the surface roughness also increases. Mainly due to the increase of cutting depth, the amount of material removed increases, and the contact stress between tool and workpiece increases, which makes the plastic deformation of the workpiece surface more intense.

## 4. Removal Mechanism of UEVC of Medical β Titanium Alloy

For UEVC machining of the surface micro texture of medical β titanium alloy, exploring the material removal mechanism in the cutting process can provide theoretical guidance for high-quality machining of the surface texture. Because the vibration frequency of the UEVC process reaches 20 kHz, it is difficult for human eyes to observe the material and chip formation mechanism in the cutting process. Therefore, based on the reproduction characteristics of the whole process of simulation, this paper explores the material removal mechanism in UEVC, including material cutting deformation and stress distribution, mechanical and thermal variation law, chip formation mechanism, and so on.

### 4.1. Analysis of UEVC Cutting Mechanism

**Figure 10:**
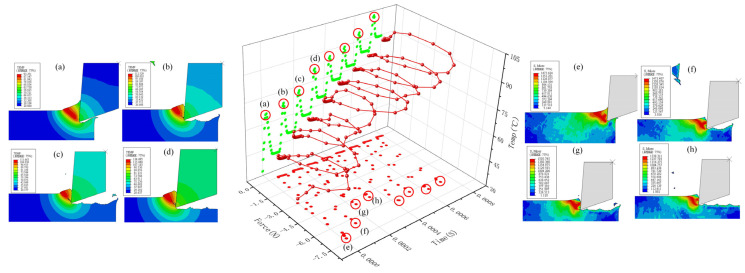
UEVC force–thermal evolution law (Temp-Cutting Temperature).

Figure 10 shows the evolution diagram of the force–thermal evolution law of the UEVC process. The circle in Figure 10 indicates the extreme points of cutting force and cutting temperature in each cutting cycle of UEVC. It can be seen from the curve in the figure that UEVC has the characteristics of tool-workpiece cycle separation. Compared with the high-temperature process at the moment of traditional cutting, the overall temperature rise trend of the UEVC process is slower. Under the simulation parameters of *v* = 140 mm/s, *f* = 20 kHz, *a_p_* = 0, *t* = 0.0008 s, the maximum temperature of the UEVC process does not exceed 120 °C;. Combined with the temperature cloud diagram and the temperature change curve, it can be seen that when the tool is not separated from the workpiece, the cutting temperature at the tool tip begins to decrease. The reason for this is that at this time, the speed of the tool along the positive direction of the Y-axis gradually increases, while it decreases along the X direction, and the contact stress between the tool and the workpiece gradually decreases. At this time, the temperature is at the maximum point of the cycle, the material plasticity is strong, and the stress state between the tool and the workpiece is weakened. The resistance of the tool when cutting the workpiece decreases, the tool–workpiece interface is about to enter the separation state, and the cutting temperature begins to decrease. Based on the temperature nephogram, the maximum temperature point in the temperature curve is extracted. It can be observed that the maximum temperature point in each cutting cycle occurs at the time at which the chip is about to break or at the beginning of fracture. At this time, the contact area and contact stress between the tool and the workpiece reach the extreme point.

At the same time, it can also be found that the cutting force has a maximum point in each cutting cycle that appears before the maximum point of cutting temperature (the stress state at the maximum point of temperature is weakened). The maximum point of cutting force in each cutting cycle appears at the greatest distance between the tool tip and the workpiece along the cutting direction, that is, with the maximum value of material removal. In the first cutting cycle, the maximum value of cutting force is greater than that of the other cutting cycles. The reason for this is that the material removal is the highest in the first cutting cycle. At this time, the cutting temperature is low, and the plasticity of the material is weak, so the bonding state between grains is strong, and the cutting resistance is large [27].

### 4.2. Analysis of Material Removal Process

**Figure 11:**
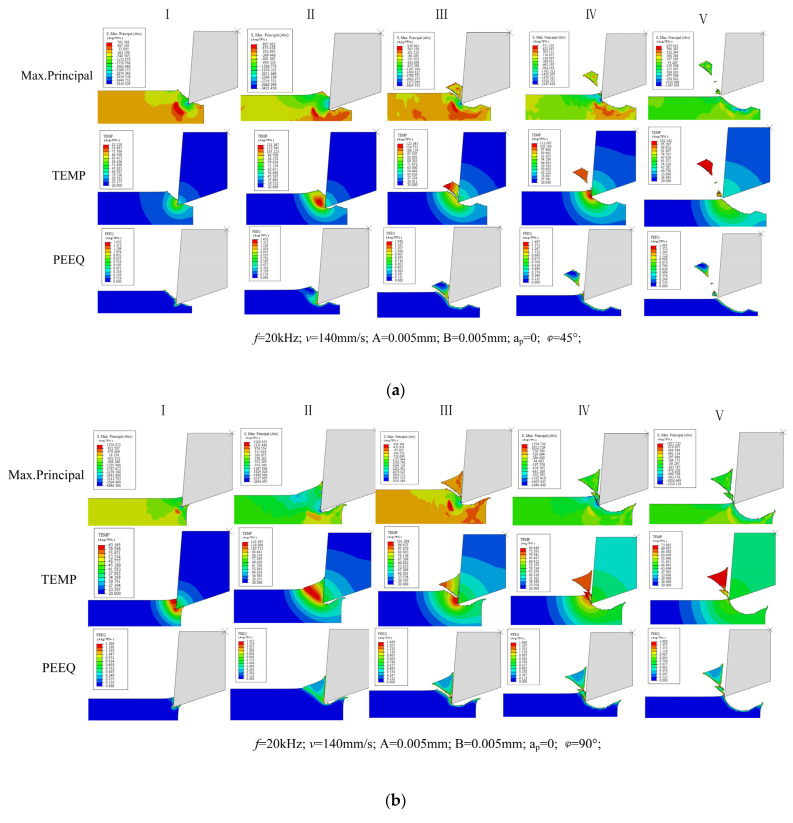

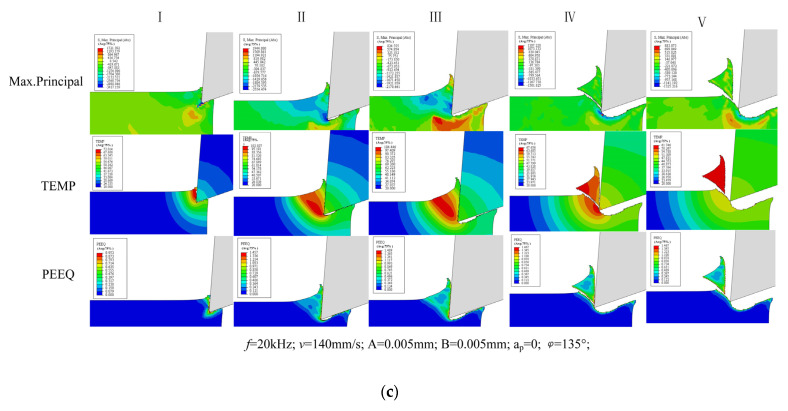
Analysis of UEVC material removal process (Max.Principal- Maximum Principal Stress, PEEQ- Plastic Strain). (**a**) Phase difference 45°; (**b**) phase difference 90°; (**c**) phase difference 135°.

The maximum principal stress, cutting temperature and plastic deformation of the material during a cutting cycle of UEVC are extracted in the simulation, as shown in Figure 11.

It is found that under different cutting conditions and vibration parameters, the material removal process of UEVC in one cutting cycle can be divided into five stages. In the first stage, the tool just cuts into the material, at this time, the material undergoes brittle fracture, and the high temperature is mainly concentrated at the tool tip. In the second stage, the tool completely cuts into the material, and the high temperature zone is located in the material shear zone in the direction of tool cutting speed. In the third stage, the chips are about to separate, and the cutting temperature begins to drop. In the fourth stage, the chips have separated, and the tool starts to cut the material again or press the material. In the fifth stage, the tool starts to exit the cutting process and the cutting cycle ends.

Figure 11a,b show the material removal mechanism of the UEVC process when the phase difference is 45° and 90°. It can be seen that the material removal methods are basically the same under the two cutting tracks. In the first stage of the cutting cycle, the tool tip just cuts into the workpiece. At this time, the cutting temperature is low, the material plasticity is weak, and there is low plastic deformation. It can be seen from the maximum principal stress diagram that the material is brittle under the extrusion of the tool. The cutting cycle enters the second stage, and the tool completely cuts into the material. At this time, the cutting heat is mainly concentrated in the material shear zone of the tool tip along the cutting speed direction. Under the influence of high temperature, the plasticity of the material is enhanced. Under the extrusion action of the cutting tool, the material produces high plastic deformation. Under the action of high temperature and tool extrusion, chip is gradually produced on the material surface, and adiabatic shear occurs. In the third stage of the cutting cycle, the vibration of the tool along the Y direction changes from negative to positive. The tool further extrudes the gradually generated chips, and the material undergoes adiabatic shear. Then the chip breaks and the fracture surface exhibits high plastic deformation, and the cutting temperature begins to drop. Through the above three stages, it can be found that the material removal process of UEVC is a state of “press–shear–pull” composite cutting. Firstly, the tool extrudes the material to preliminarily form chips. A shear force is then applied to the formed chip. The plastic deformation at the chip fracture indicates that a certain tensile stress is generated on the surface during the fracture. Under this cutting path in Figure 11a,b, the machined surface produces certain residual protrusions under the action of the tensile stress of the chip. The tool will then perform secondary cutting on the machined surface. In the fourth stage of cutting, the tool continues to vibrate upward. The vibrating cutter carries out secondary cutting on the machined surface and produces certain chips to make the machined surface more flat. In the fifth stage of cutting, the tool starts to exit the cutting state and enter the empty cutting stage of a cutting cycle. At this stage, the tool and material are separated. At this time, the cutting area is opened, and the lubricating fluid can fully lubricate the cutting area.

Figure 11c shows the material removal mechanism of the UEVC process when the phase difference is 135°. Due to the difference of cutting path, in the first stage, the heat at the tool tip is mainly concentrated on the front face of the tool, and the heat at the back face is small. In addition, different from the UEVC process when the phase difference is 45°and 90°, there is no secondary cutting in the cutting process when the phase difference is 135°. Only one chip is produced in the whole cutting cycle, as can be seen from the analysis of the cutting trajectory in Figure 3. When the phase difference is 135°, the cutting speed of the tool along the X direction changes from negative to positive, which is earlier than the transition node in the Y direction. The tool exits the cutting state in advance. The secondary cutting of the machined surface is transformed into ironing. Different from the residual tensile stress produced by secondary cutting, there is a high residual compressive stress on the material surface. During the second stage, it can be seen that under this cutting trajectory, the compressive stress distribution in the material shear zone is more obvious. Combined with the temperature nephogram, it is found that the cutting temperature at this time is significantly lower than that at phase differences of 45° and 90°. Therefore, the plasticity of the material is weak, and the shear force required for material removal is greater.

**Figure 12:**
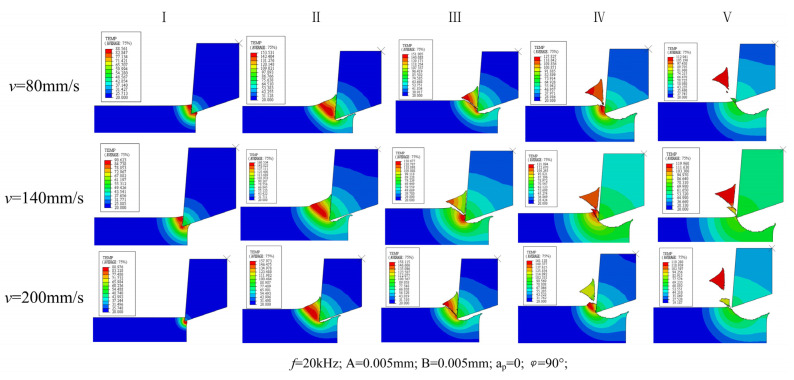
Material removal process at different cutting speeds.

Figure 12 shows the temperature nephogram of UEVC material removal process at different cutting speeds. It can be seen from the figure that there is no significant difference in the material removal process when the cutting speed increases from 80 mm/s to 200 mm/s. It is consistent with the removal method under the phase difference of 90° analyzed above. From the temperature nephogram, it can be seen that the cutting temperature changes significantly under different cutting speeds.

Based on the above analysis, it can be concluded that the difference in cutting trajectory under phase difference control has a significant impact on the material removal mode. The influence of cutting speed on material removal is not obvious.

### 4.3. Surface Machining Quality Analysis of UEVC

**Figure 13:**
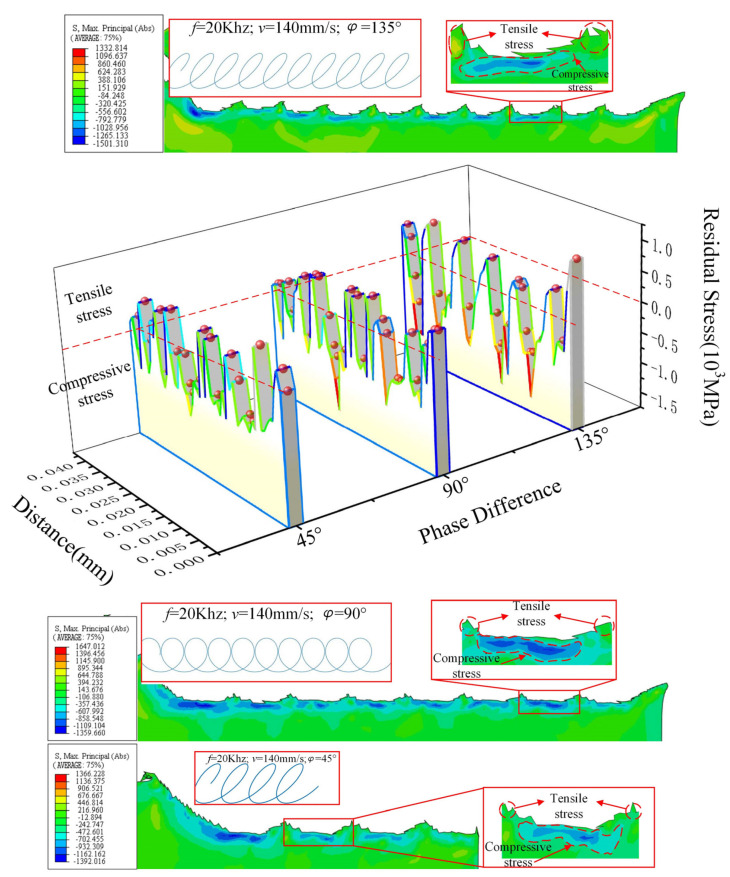
Evolution of surface residual stress of UEVC material.

The residual stress of UEVC machined surface with phase differences of 45°, 90° and 135° is extracted to obtain the evolution diagram of residual stress on the UEVC material surface, as shown in Figure 13.

Through the analysis of the machined surface topography under the two cutting trajectories in Figure 13, it can be obtained that different cutting trajectories will produce different morphologies on the material surface. For machining of different surface topographies, different cutting trajectories can be generated by adjusting the cutting parameters and ultrasonic parameters. It can be found from the curve that under the two cutting tracks, the residual stress on the machined surface is mainly compressive stress, but the tensile stress is also intermittently distributed. In addition, the tensile stress distribution range is wide when the phase difference is 135°. Compared with the surface residual stress diagram, it can be found that the tensile stress appears at the surface protrusion position, that is, the position where one effective cutting cycle (tool–workpiece contact) ends and another effective cutting cycle begins. The reason for this is that in the later stage of the effective cutting cycle, the tool begins to pull the material upward, resulting in tensile stress at the protrusion position [28]. In the next cutting cycle, the tool cuts the material using tensile stress, resulting in a small distribution range of tensile stress on the machined surface. Compared with the phase difference of 45° and 90°, the tensile stress distribution range of machined surface with 135° phase difference is wider. By analyzing the cutting path when the phase difference is 135°, it is found that the lifting stage of the tool is long.

**Figure 14:**
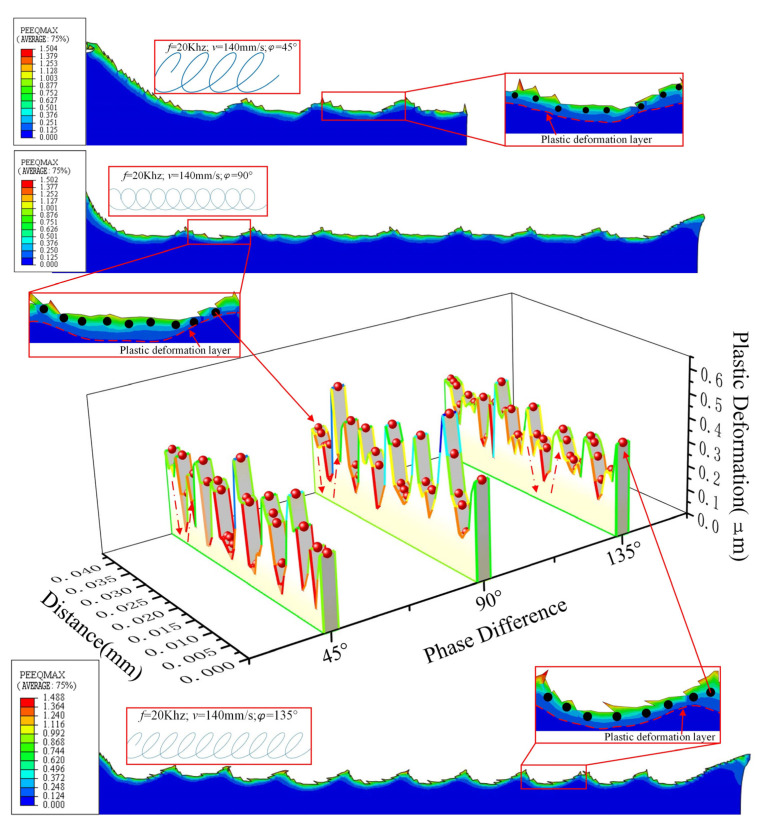
Evolution of surface plastic deformation of UEVC materials.

The plastic deformation value of the UEVC machined surface is extracted to obtain the evolution diagram of plastic deformation of the UEVC material surface, as shown in Figure 14. By analyzing the plastic deformation of the machined surface, it can be concluded that the degree of plastic deformation of the machined surface with phase difference of 45° and 90° is higher than that with a phase difference of 135°. In a cutting cycle under the two cutting trajectories, the plastic deformation value of the machined surface first decreases and then increases. At the same time, it can be found that the thickness of plastic deformation layer first increases and then decreases during a cutting cycle. The main reason for this is that there is compressive stress at the lowest point of the machined surface, and the stress transmission range is wider [29]. It leads to a wider range of plastic deformation along the depth direction. The reason the plastic deformation value is opposite to the thickness of the deformation layer is the different effect of stress [30]. At the beginning of a cutting cycle, the tool cuts downward, and the material surface bears the tensile stress in the cutting direction. Therefore, high plastic deformation occurs along the cutting direction, but the effect of compressive stress along the depth direction is weak. When the tool is near the lowest point, the material surface mainly bears the compressive stress along the depth direction, which is mainly manifested in the greater depth of plastic deformation. The different performance of the two trends further shows that there is a “press–shear–pull” transformation in the removal process of UEVC material.

## 5. Conclusions

In this paper, a UEVC finite element simulation model was established, and the UEVC cutting trajectory is planned. The cutting mechanism of the UEVC process is explored through simulation analysis. Through the analysis of cutting deformation, stress distribution, force heat change law and chip formation mechanism in each stage of the UEVC process, the mechanism of micro removal of materials in the UEVC process was obtained. The results show that:

(1) The cutting temperature and cutting force in the UEVC process follow a law of periodic change. Different from traditional cutting, the cutting temperature in the UEVC process has a decreasing stage. The maximum point of cutting force in each cutting cycle is when the material removal is the greatest. The maximum point of cutting force is ahead of the maximum point of cutting temperature.

(2) The removal process of UEVC material is a “press–shear–pull” composite cutting process. When the tool cuts into the material, the material is brittle. With increasing temperature, the material undergoes plastic deformation. The tool extrudes the material to form the chips, and the material is removed by adiabatic shear under the influence of high temperature.

(3) Differences in UEVC trajectories affect the removal mode of materials and form different surface morphologies. In a cutting cycle, when the phase difference is 45° and 90°, there is secondary cutting in the cutting process. When the phase difference is 135°, the tool presses the location of chip separation following chip separation. Both methods make the machined surface more flat.

(4) For different cutting paths, compressive stress is distributed at the lowest point of the machining pit, and tensile stress is distributed at the protrusion position. The plastic deformation value of the surface layer of the machined surface first decreases and then increases, and the thickness of the surface plastic deformation layer first increases and then decreases. Compared with phase differences of 45° and 90°, the distribution range of compressive stress and plastic deformation of machined surface at 135° phase difference are smaller.

## Figures and Tables

**Table 1:** Workpiece parameter settings.

Parameter	Number
*A* (MPa)	1098
*B* (MPa)	1092
*n*	0.93
*C*	0.014
*m*	1.1
$T_{r}$ (°C)	20
$T_{m}$ (°C)	1680
*d*_1_	−0.09
*d*_2_	0.25
*d*_3_	−0.5
*d*_4_	0.014
*d*_5_	3.87
*K*	7
*C*	546,000,000
$\varepsilon_{0}$	1
μ	0.3

**Table 2:** Cutting parameter setting.

Group	Cutting Speed *V_f_* (m/min)	Feed Rate *F* (μm/r)	Cutting Depth *a*_*p*_ (μm)	Ultrasonic Amplitude P-P (μm)	Ultrasonic Frequency (kHz)
**1**	1	20	6	4	20
**2**	1	25	6	4	20
**3**	1	20	3	4	20
